# GLP-1 Analogue-Loaded Glucose-Responsive Nanoparticles as Allies of Stem Cell Therapies for the Treatment of Type I Diabetes

**DOI:** 10.1021/acsptsci.4c00173

**Published:** 2024-05-02

**Authors:** Joana Moreira Marques, Rute Nunes, Ana Margarida Carvalho, Helena Florindo, Domingos Ferreira, Bruno Sarmento

**Affiliations:** † i3S—Instituto de Investigação e Inovação em Saúde, Universidade do Porto, 4200-135 Porto, Portugal; ‡ INEB—Instituto de Engenharia Biomédica, Universidade do Porto, Rua Alfredo Allen, 208, 4200-180 Porto, Portugal; § UCIBIO—Applied Molecular Biosciences Unit, REQUIMTE, MedTech–Pharmaceutical Technology Laboratory, Drug Sciences Department, Faculty of Pharmacy, University of Porto, 4099-002 Porto, Portugal; ∥ IUCS-CESPU - Instituto Universitário de Ciências da Saúde, 4585-116 Gandra, Portugal; ⊥ ICBAS—Instituto de Ciências Biomédicas Abel Salazar, Universidade do Porto, Rua de Jorge Viterbo Ferreira, 228, 4050-313 Porto, Portugal; # Research Institute for Medicines (iMed.ULisboa), Faculty of Pharmacy, Universidade de Lisboa, Av. Prof. Gama Pinto, 1649-003 Lisbon, Portugal

**Keywords:** stem cell therapy, diabetes, GLP-1 analogues, glucose-responsive nanoparticle, bioartificial pancreas

## Abstract

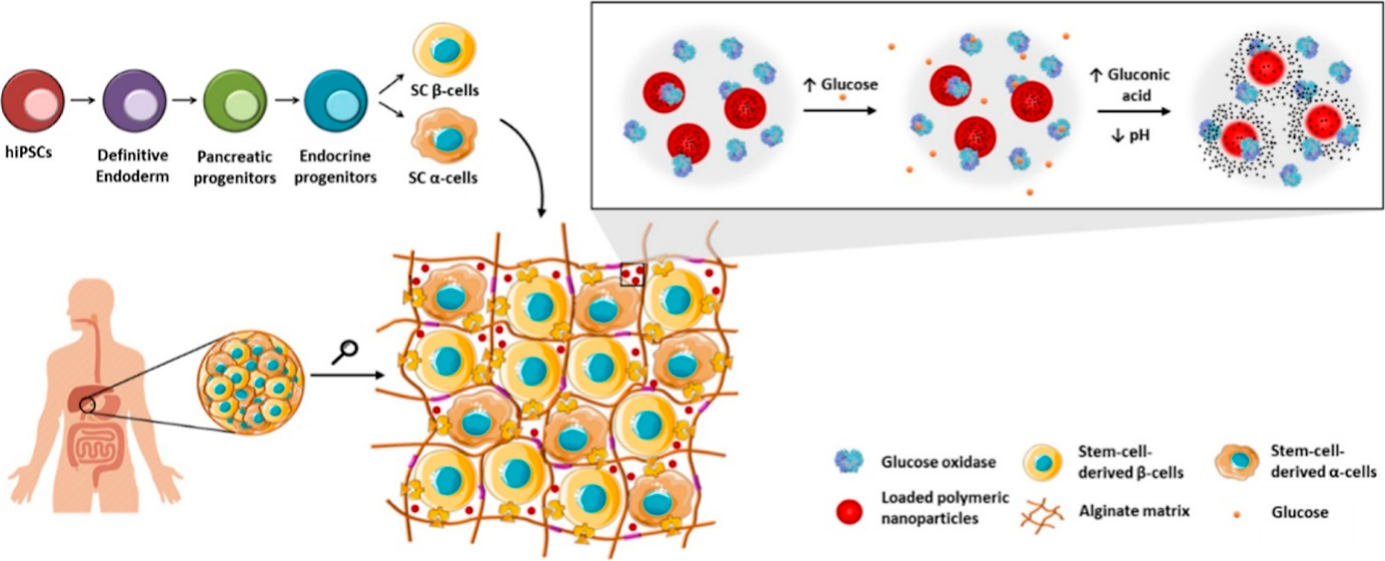

Type 1 diabetes (T1D) is characterized by insufficient insulin secretion due to β-cell loss. Despite exogenous insulin administration being a lifesaving treatment, many patients still experience severe glycemic lability. For these patients, a β-cell replacement strategy through pancreas or pancreatic islet transplantation is the most physiological approach. However, donors’ scarcity and the need for lifelong immunosuppressive therapy pose some challenges. This study proposes an innovative biomimetic pancreas, comprising β- and α-cells differentiated from human induced pluripotent stem cells (hiPSCs) embedded in a biofunctional matrix with glucose-responsive nanoparticles (NPs) encapsulating a glucagon-like peptide 1 (GLP-1) analogue, which aims to enhance the glucose responsiveness of differentiated β-cells. Herein, glucose-sensitive pH-responsive NPs encapsulating exenatide or semaglutide showed an average size of 145 nm, with 40% association efficiency for exenatide-loaded NPs and 55% for semaglutide-loaded NPs. Both peptides maintained their secondary structure after in vitro release and showed a similar effect on INS-1E cells’ insulin secretion. hiPSCs were differentiated into β- and α-cells, and insulin-positive cells were obtained (82%), despite low glucose responsiveness, as well as glucagon-positive cells (17.5%). The transplantation of the developed system in diabetic mice showed promising outcomes since there was an increase in the survival rate of those animals. Moreover, diabetic mice transplanted with cells and exenatide showed a decrease in their glucose levels. Overall, the biomimetic pancreas developed in this work showed improvements in diabetic mice survival rate, paving the way for new cellular therapies for T1D that explore the synergy of nanomedicines and stem cell-based approaches.

Diabetes mellitus (DM) includes a group of chronic metabolic diseases characterized by high blood glucose levels due to deficiencies in insulin secretion, insulin action (or insulin resistance), or both.^1^ Type 1 diabetes (T1D) is characterized by autoimmune destruction of insulin-producing β-cells in the pancreatic islets of Langerhans cells, resulting in insulin depletion.^2^ The disease has an early onset, being frequently diagnosed during childhood, and it is estimated that there were around 8.4 million individuals with T1D worldwide in 2021.^3^ Typically, T1D treatment consists of exogenous insulin administration for glycemic control, either through multiple daily injections or through sensor-augmented insulin pumps.^4^ Despite greatly improving patients’ quality of life, exogenous insulin does not allow for an efficient control of blood glucose levels. This limitation stems from its inability to fully compensate for β-cell loss and also from the complex interplay of factors affecting glucose control besides insulin, such as diet, metabolism, and physical activity.^5,6^ Therefore, diabetes management may be better achieved when a more physiological approach is adopted, in which the pancreas secretes a basal low amount of insulin and a higher amount when glucose levels increase after a meal.

When T1D patients show poor glycemic control, with frequent hyper- and hypoglycemic events and associated complications, even when under exogenous insulin therapy, transplantation of whole-pancreas or pancreatic islets offers an alternative to provide long-lasting exogenous insulin independence.^7^ Nevertheless, there are undoubtedly drawbacks associated with these procedures, mainly donors’ scarcity, the need for immunosuppression to avoid graft rejection, and the decrease in islet graft survival and functionality over time.^8^ Hence, alternative strategies have been explored in the past decades for the treatment of T1D. Of note, the differentiation of human pluripotent stem cells into pancreatic cell types could be an extremely valuable solution to achieve an improved and personalized therapy for this disease. However, differentiation protocols developed so far still originate immature cells, which lack full glucose responsiveness when compared to adult human pancreatic islets.^9^

In this work, we propose the development of an innovative biomimetic pancreas comprising beta and alpha cells differentiated from human induced pluripotent stem cells (hiPSCs) and immobilized in a biofunctional matrix, embedding glucose-responsive nanoparticles (NPs) encapsulating a glucagon-like peptide 1 (GLP-1) analogue. The incorporation of a GLP-1 analogue in the system aims to stimulate the glucose responsiveness of differentiated beta cells since they are still immature and not fully responsive. The glucose-dependent release will be accomplished by the incorporation of glucose oxidase (GOx) into the system. In the presence of high levels of glucose, GOx degrades this monosaccharide into gluconic acid, decreasing the surrounding pH (∼5). Engineered pH-sensitive NPs will respond to a decrease in the environmental pH by releasing their payload. Two GLP-1 analogues were chosen for this study: exenatide (EXN) and semaglutide (SMG). EXN is a synthetic derivative of exendin 4, a peptide isolated from salivary secretions of the Gila monster lizard (*Heloderma suspectrum*), presenting 53% amino acid sequence homology with GLP-1 and a higher binding affinity to GLP-1 receptor (GLP-1R) compared to GLP-1.^10,11^ EXN exerts glucoregulatory effects similar to those of GLP-1 that include glucose-dependent stimulation of insulin secretion, suppression of glucagon secretion, slowing of gastric emptying, increased insulin sensitivity, and promotion of β-cell proliferation.^11,12^ On the other hand, SMG is 94% homologous to the native GLP-1, having modifications at the lysine residue in the 34th position, the alanine residue in the eighth position to protect SMG from DPP-4 enzymatic degradation, and the lysine residue in the 26th position where a C18 fatty diacid was attached through a hydrophilic linker to prolong SMG systemic half-life.^13,14^

## Results and Discussion

As a potential treatment or cure for T1D and with the goal to surpass some of the problems of the current treatments available, we developed a biomimetic pancreas system comprising differentiated pancreatic α- and β-cells from hiPSCs, GLP-1 analogue-loaded glucose-responsive nanoparticles, and GOx coencapsulated in an alginate matrix. GOx was included in the system in order to achieve glucose-dependent peptide release from NPs. GOx catalyzes the oxidation of β-d-glucose to d-glucono-δ-lactone and hydrogen peroxide in the presence of molecular oxygen. d-glucono-δ-lactone is subsequently hydrolyzed by lactonase to d-gluconic acid,^15^ thereby transiently decreasing the surrounding pH. By incorporating a pH-sensitive polymer in the nanoformulation and GOx in the system, the peptide release can be tuned according to glucose levels.

Given the complexity of the system, it was developed by dividing it into different steps, starting with the development of the NPs, followed by the generation of stem-cell-derived (SC−) β-cells and α-cells, and then the encapsulation of all the components.

### Characterization of Nanoparticles

GLP-1 analogue-loaded NPs were incorporated into the system to improve glucose-stimulated insulin secretion of SC-β-cells since they are not fully mature and functional after in vitro differentiation.

To achieve peptide release in a pH-dependent manner, Eudragit E100 was included in the NP formulation, besides PLGA. Different ratios of both polymers were tested (data not shown) until the final formulation was achieved. Peptide-loaded pH-sensitive NPs were successfully produced by a modified solvent emulsification-evaporation method based on the water-in-oil-in-water (w1/o/w2) double emulsion technique, as previously described.^16^

**Figure 1:**
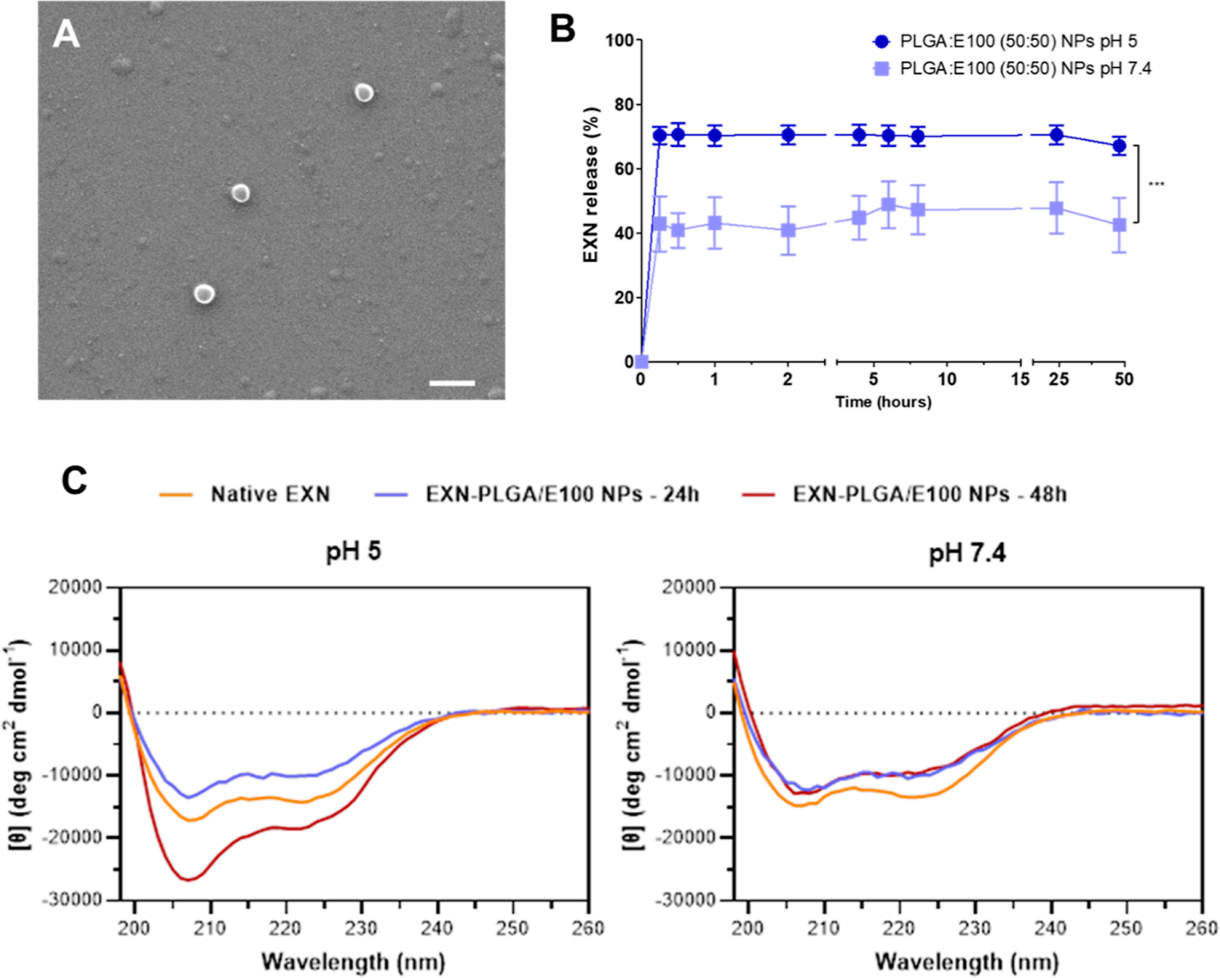
Morphological features and drug release profile of EXN-loaded NPs. (A) SEM image of EXN-loaded PLGA:E100 NPs (50:50) (scale bar: 500 nm). (B) Release profile of EXN from NPs at pH 5 and pH 7.4. Points and vertical bars stand for mean and standard deviation (SD), respectively, and (***) indicates *p* < 0.001 (*n* = 3). (C) Far UV CD spectra for EXN released from NPs during the in vitro release assay at pH 5 (left) and 7.4 (right). Freshly prepared EXN solution (native EXN) was used as a reference. EXN-PLGA/E100 NPs denote EXN-loaded PLGA:E100 (50:50) NPs.

**Figure 2:**
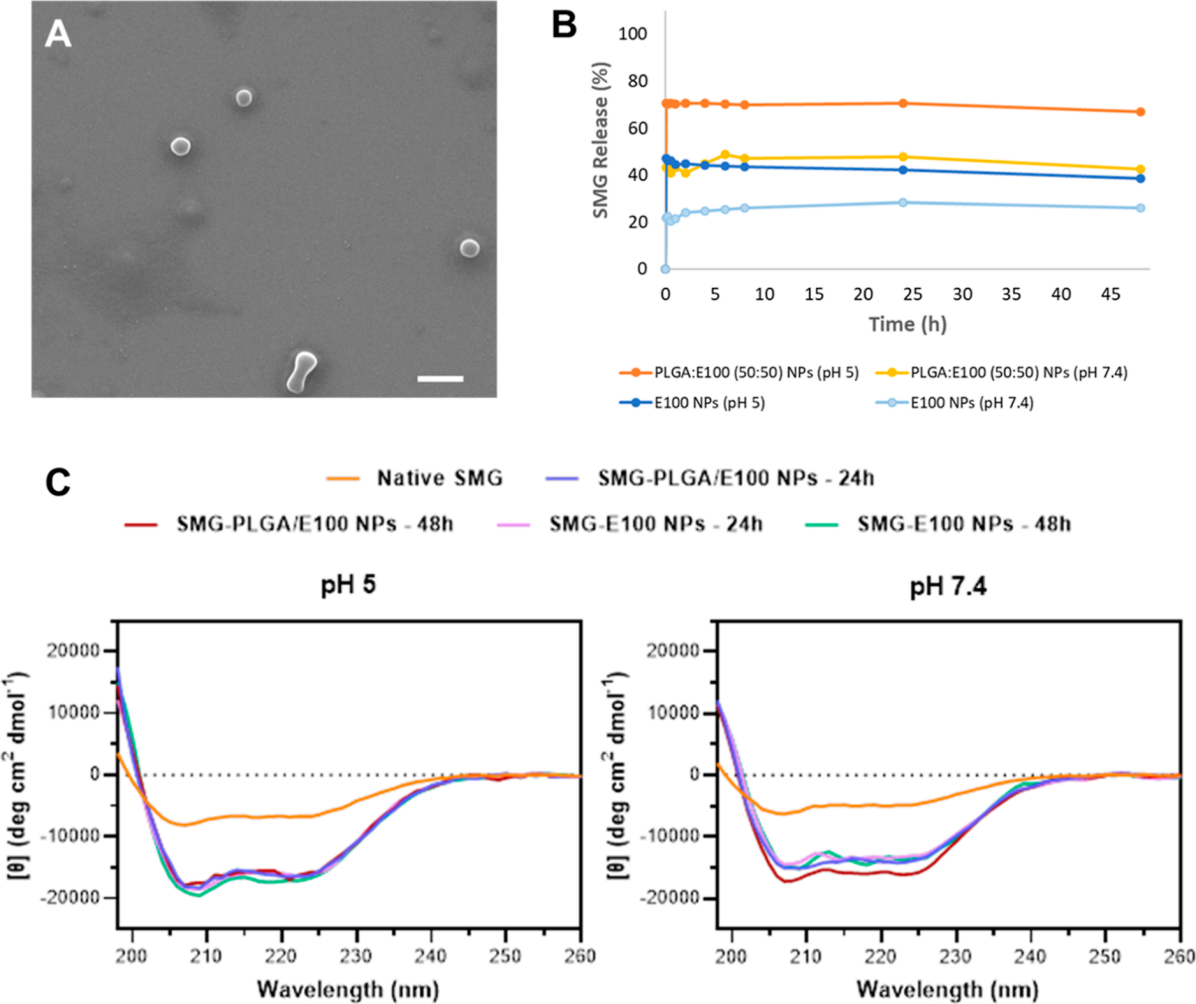
Morphological features and drug release profile of SMG-loaded NPs. (A) SEM image of SMG-loaded PLGA:E100 NPs (50:50) (scale bar: 500 nm). (B) Release profile of SMG from NPs at pH 5 and pH 7.4. Points and vertical bars stand for mean and SD, respectively, and (***) indicates *p* < 0.001 (*n* = 3). (C) Far UV CD spectra for SMG released from NPs during in vitro release assay at pH 5 (left) and 7.4 (right). Freshly prepared SMG solution (native SMG) was used as a reference. SMG-PLGA/E100 NPs denote SMG-loaded PLGA:E100 (50:50) NPs, and SMG-E100 NPs denote SMG-loaded E100 NPs.

In this work, two peptides were explored, namely, EXN and SMG. For each peptide, two formulations [PLGA:E100 (50:50) and E100] and three drug loadings (5, 10, and 15%) were tested and characterized regarding size, polydispersity index (PDI), and zeta potential (ZP) (Table 1). The results for SMG-loaded E100 NPs with 15% of theoretical drug loading are not shown in Table 1 since this formulation showed some aggregates. All formulations presented a mean size around 135–155 nm and a monodisperse population (PDI < 0.200), as well as a ZP between 17 and 42 mV, which decreased with increasing amounts of loaded peptide. This is due to the negative charge of both EXN [isoelectric point (pI): 4.86] and SMG (pI: 5.4) at pH 7, which interact with the positively charged E100 through electrostatic forces. SEM images revealed the spherical shape of NPs and confirmed the size obtained by dynamic light scattering (DLS) (Figure 1A; Figure 2A).

**Table 1:** Physicochemical Properties of EXN- and SMG-Loaded NPs Produced by the Double Emulsion Technique^a^

drug	theoretical drug loading	formulation	size (nm)	PDI	ZP (mV)	AE (%)	DL (%)
EXN	5%	PLGA:E100 (50:50)	149.4 ± 3.7	0.163 ± 0.016	31.1 ± 0.7	47.4 ± 6.3	2.4 ± 0.3
		E100	135.9 ± 5.4	0.155 ± 0.012	42.1 ± 1.1	49.0 ± 5.1	2.4 ± 0.3
	10%	PLGA:E100 (50:50)	140.9 ± 2.5	0.164 ± 0.009	29.2 ± 1.2	41.4 ± 0.9	4.1 ± 0.1
		E100	134.8 ± 2.5	0.155 ± 0.016	25.8 ± 5.1	34.2 ± 1.1	3.4 ± 0.1
	15%	PLGA:E100 (50:50)	146.0 ± 2.6	0.170 ± 0.017	21.7 ± 2.7	33.9 ± 0.8	5.1 ± 0.1
		E100	138.5 ± 4.0	0.167 ± 0.014	17.6 ± 4.2	36.9 ± 0.7	5.5 ± 0.1
SMG	5%	PLGA:E100 (50:50)	149.7 ± 5.7	0.177 ± 0.016	29.3 ± 2.9	64.0 ± 2.8	3.2 ± 0.1
		E100	135.8 ± 3.5	0.154 ± 0.010	28.5 ± 4.0	60.1 ± 3.2	3.0 ± 0.2
	10%	PLGA:E100 (50:50)	146.6 ± 5.1	0.165 ± 0.013	25.7 ± 2.9	50.1 ± 13.9	5.0 ± 1.4
		E100	153.9 ± 4.3	0.173 ± 0.016	25.1 ± 4.1	52.8 ± 2.4	5.3 ± 0.2
	15%	PLGA:E100 (50:50)	153.1 ± 4.6	0.163 ± 0.011	16.9 ± 3.6	36.8 ± 2.3	8.5 ± 0.3

a Results are presented as mean ± standard deviation (*n* = 3). Abbreviations: polydispersity index (PDI), zeta potential (ZP), association efficiency (AE), and drug loading (DL).

The incorporation of a pH-responsive polymer (Eudragit E100) in the NPs composition was used to modify the release profile of the loaded peptide, thereby increasing the release of their cargo when the optimum pH window is reached. Considering the aim of the work, E100 was chosen because it starts to solubilize at pH 5 or below. This pH is achieved by the increased amounts of gluconic acid produced from glucose oxidation by GOx, producing holes in the NP matrix that accelerate the release of the preloaded drug.^17^ The association efficiency (AE) and drug loading (DL) of EXN were slightly lower than those of SMG in all cases when comparing the same theoretical drug loading and formulation (Table 1). This could be explained by the presence of an 18-carbon fatty acid moiety in the SMG structure, which confers a higher degree of hydrophobicity, thus promoting the interaction between SMG and the polymers.

The release profiles of EXN and SMG were evaluated in vitro over 48 h. As expected, PLGA:E100 NPs (50:50) displayed a pH-dependent in vitro release profile, releasing around 70% of the loaded EXN at pH 5, while this amount was only 40% when NPs were exposed to pH 7.4 (Figure 1B). Similarly, SMG-loaded NPs released 15 to 20% more peptide in acidic conditions when compared to pH 7.4, depending on the formulation (Figure 2B). This contrasts with the lack of pH-responsiveness behavior of PLGA:E100 (90:10) NPs when tested in the same conditions, since these NPs have a significantly lower amount of E100 in their composition (Figure S1). Although a burst release of EXN and SMG was observed within the first hour at both pH conditions, likely due to the leaching of the surface-adsorbed peptide, it was followed by a slow release up to at least 48 h.

Circular dichroism (CD) spectroscopy analysis was performed to evaluate the secondary structure of each peptide after its in vitro release from NPs. Samples were collected at 24 and 48 h after the beginning of the assay at each pH condition (5 and 7.4). The results obtained reveal that there was no significant change in the secondary structure of both peptides after encapsulation and throughout the in vitro release study in all tested conditions since the CD spectra profile is maintained when compared to the native peptide (Figures 1C and 2C). Results show that both EXN and SMG have a characteristic α helix profile, with two negative peaks of similar magnitude at 222 and 208 nm,^18^ as expected and similar to GLP-1 structure.^14,19^ Although not significant, there was a slight change in the magnitude of the peak at 208 nm for EXN collected 48 h after release from NPs at pH 5. It is reported that the relative magnitudes of the peaks at 222 and 208 nm may vary depending on H-bonding and the hydrophobicity of the environment.^18^ Nevertheless, considering the physiologic conditions in which the complete system will be, the peptide will not be exposed to an acidic environment for 48 consecutive hours but rather to intermittent variations of pH (between 7.4 and 5, depending on the glucose levels). Taking this into account and the physiologic conditions of the target application, both peptides can be considered to be stable for at least 48 h after encapsulation.

### Glucoregulatory Effect of EXN and SMG on INS-1E Cells

**Figure 3:**
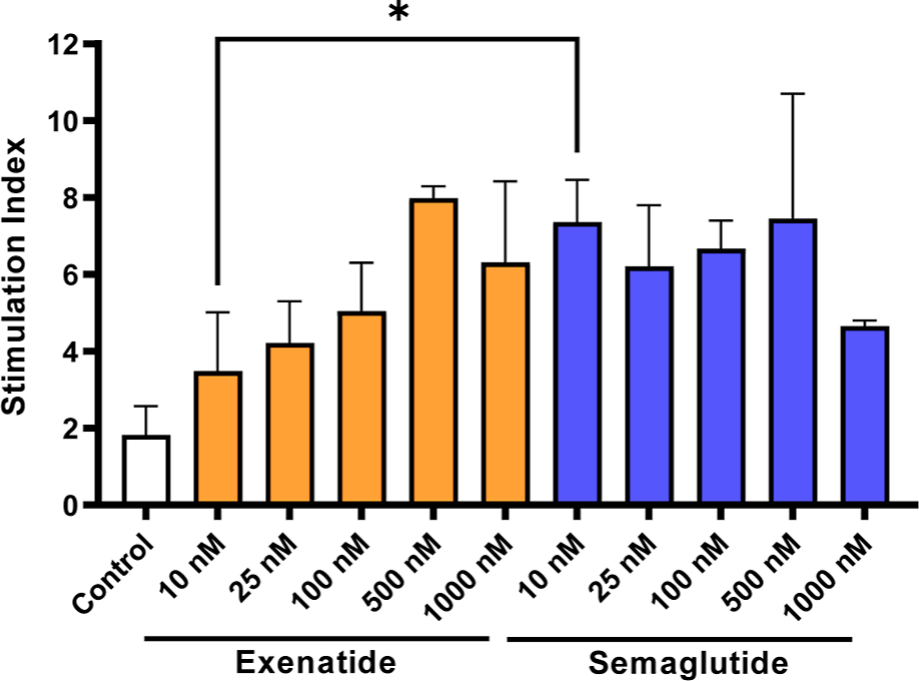
SI after the exposure of INS-1E cells to GLP-1 analogues (EXN or SMG). Insulin secretion from INS-1E cells in response to low (2 mM) and high (20 mM) glucose concentrations in the presence of different concentrations of either EXN or SMG. Secreted insulin was quantified by ELISA. Bars represent the mean, and error bars represent the standard deviation.

The inclusion of a GLP-1 analogue in the system intends to boost insulin secretion by SC-β-cells in response to glucose. The presence of glucose-sensitive NPs ensures the release and action of the GLP-1 analogue when high glucose levels are present. In order to determine which drug to include in the system and also the best drug concentration, a glucose-stimulated insulin secretion assay was performed with the rat insulinoma cell line INS-1E (Figure 3). To the best of our knowledge, there are no previous in vitro studies comparing the insulinotropic effect of each drug on INS-1E cells under the same conditions. Previous studies have been performed separately for EXN and SMG with different experimental conditions,^20,21^ which would make comparison between both peptides difficult.

Cells were incubated under low (2 mM) or high (20 mM) glucose concentrations, and the amount of insulin released was determined by ELISA (Figure 3). There was no striking difference between the two peptides (except for 10 nM concentration), which could be attributed to their shared mechanism of action, despite having different potencies [EC_50_: 0.11 nM (EXN) and 0.15 nM (SMG)].^22^ A steady increase in insulin secretion is observed with increasing concentrations of EXN, while SMG does not follow the same trend and shows a similar stimulation index (SI) across the different concentrations tested. Taking these results into account and that EXN stimulates stem cell differentiation into β-cells,^23−25^ this peptide was chosen to proceed with our studies.

### Generation of Pancreatic Cells from hiPSCs

**Figure 4:**
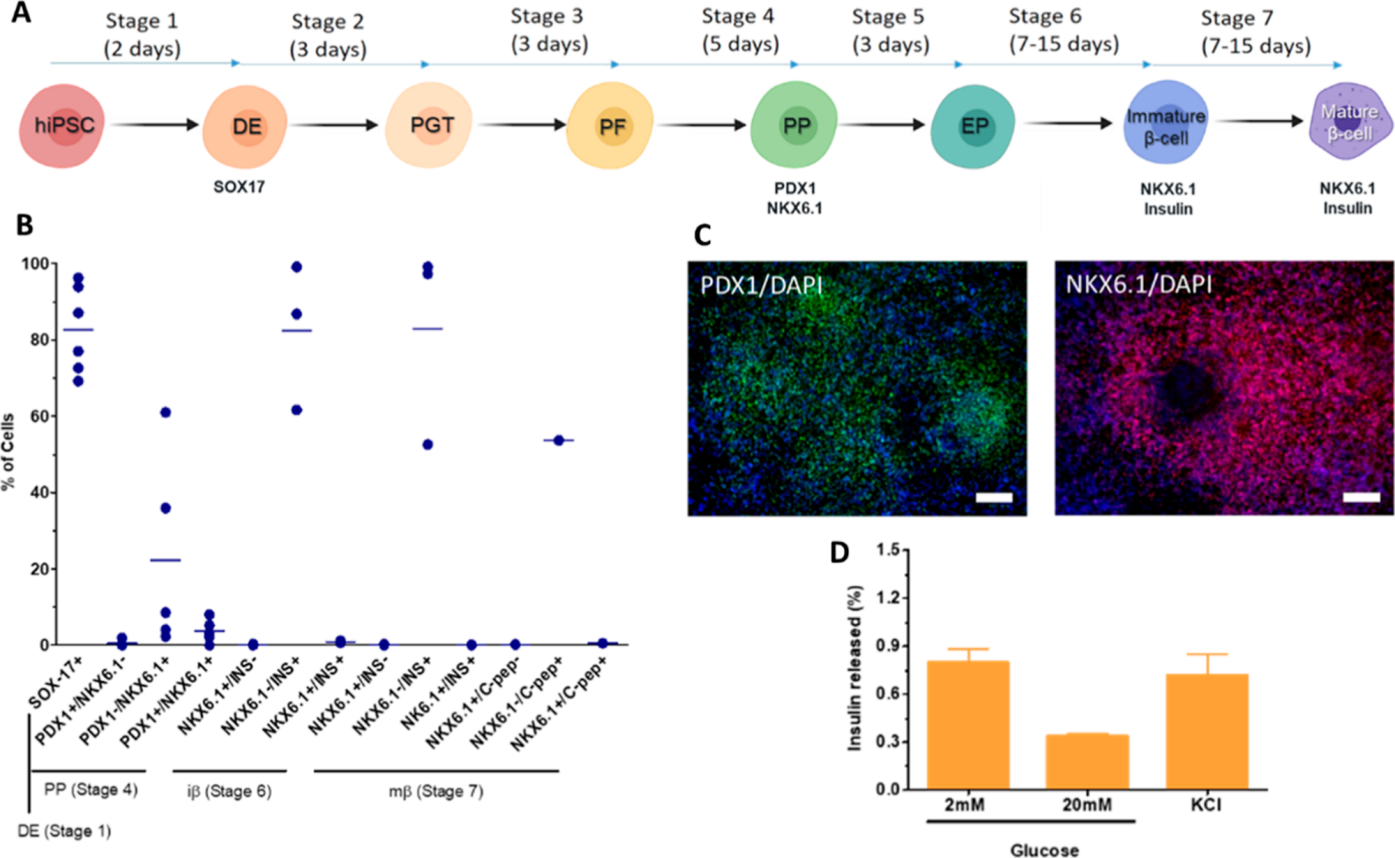
β-Cell differentiation from the WLS-4D1 hiPS cell line. (A) Schematic representation of the differentiation strategy for the stepwise generation of pancreatic β-cells. Key markers of the differentiation stages that were quantified by flow cytometry are represented below. Legend: DE, definitive endoderm; EP, endocrine progenitors; hiPSC, human induced pluripotent stem cell; iβ, immature β-cells; mβ, mature β-cells; NKX6.1, Nirenberg and Kim homeobox 6.1; PDX1, pancreatic and duodenal homeobox factor 1; PF, posterior foregut; PGT, primitive gut tube; PP, pancreatic progenitors; and SOX17, sex-determining region Y-box 17. (B) Summary of flow cytometry analysis of several differentiation stage markers (*n* = 1–6). (C) Representative immunofluorescence images of pancreatic progenitors PDX1 and NKX6.1 markers. PDX1 in green, NKX6.1 in red, and DAPI in blue. Scale bar: 100 μm. (D) Glucose-stimulated insulin secretion (GSIS) by SC-β-cells (Stage 7). A first incubation period in a 2 mM (low) glucose solution in Krebs buffer was followed by a second incubation period in a 20 mM (high) glucose solution in Krebs buffer. A depolarization challenge was performed by incubating cells with KCl. Secreted and intracellular insulin were quantified by ELISA.

hiPS cell line WLS-4D1 was differentiated into β-cells following a stepwise protocol (Figure 4A). The success of differentiation was confirmed by assessing the expression of specific markers at key differentiation stages by flow cytometry (Figure 4B). At the end of the definitive endoderm (DE) stage, the vast majority (between 72 and 96%) of differentiating WLS-4D1 cells expressed the sex-determining region Y-box 17 (SOX17). This result indicates a high commitment to the definitive endoderm lineage,^26^ essential for β-cells differentiation. However, at the end of the pancreatic progenitors (PP) stage, the maximum percentage of Nirenberg and Kim homeobox 6.1 (NKX6.1) and pancreatic and duodenal homeobox factor 1 (PDX1) coexpression obtained in 5 independent experiments was only around 11% (Figure 4B,C). Both transcription factors (TFs) are reported to be markedly expressed in PP cells and functional insulin-secreting β-cells. Evidence shows that PDX1 and NKX6.1 coexpression is crucial for the generation of glucose-responsive monohormonal β-cells^27,28^ and, specifically, NKX6.1 has a key role in β-cell maturation and functionality.^27,29^ Therefore, the low coexpression of PDX1 and NKX6.1 observed could compromise further stages of differentiation. Nevertheless, variability in differentiation efficiency is reported for several protocols and cell lines^30^ and depends on the characteristics of the iPSC cell line and the robustness of the differentiation protocol.

At the end of stage 4, differentiated PP cells were transferred to an air–liquid interface to proceed to the next differentiation stages.^31^ At the end of Stage 6 and Stage 7 (days 25 and 32, respectively), insulin expression was above 50% with a low expression of NKX6.1 (Figure 4B). This result (insulin-positive, NKX6.1-negative) indicates a polyhormonal population of immature β-cells and is reported in several in vitro protocols of β-cells differentiation.^27,29,32^

The main difference between immature and mature β-cells is their ability to respond to glucose challenges in vitro.^31^ Therefore, the ability of the SC-β-cells to produce and release insulin in response to glucose was assessed in a glucose-stimulated insulin secretion (GSIS) assay (Figure 4, part D). Differentiated β-cells showed insulin secretion, thereby confirming the presence of insulin secretory granules. However, this occurred in a variable and independent manner, since the amount of insulin released was lower when cells were exposed to a high glucose concentration (20 mM) than to a low glucose concentration (2 mM). This behavior is attributed to the low expression of NKX6.1 observed in stages 6 and 7 (Figure 4B), typically reported for polyhormonal cells.^27,29^

**Figure 5:**
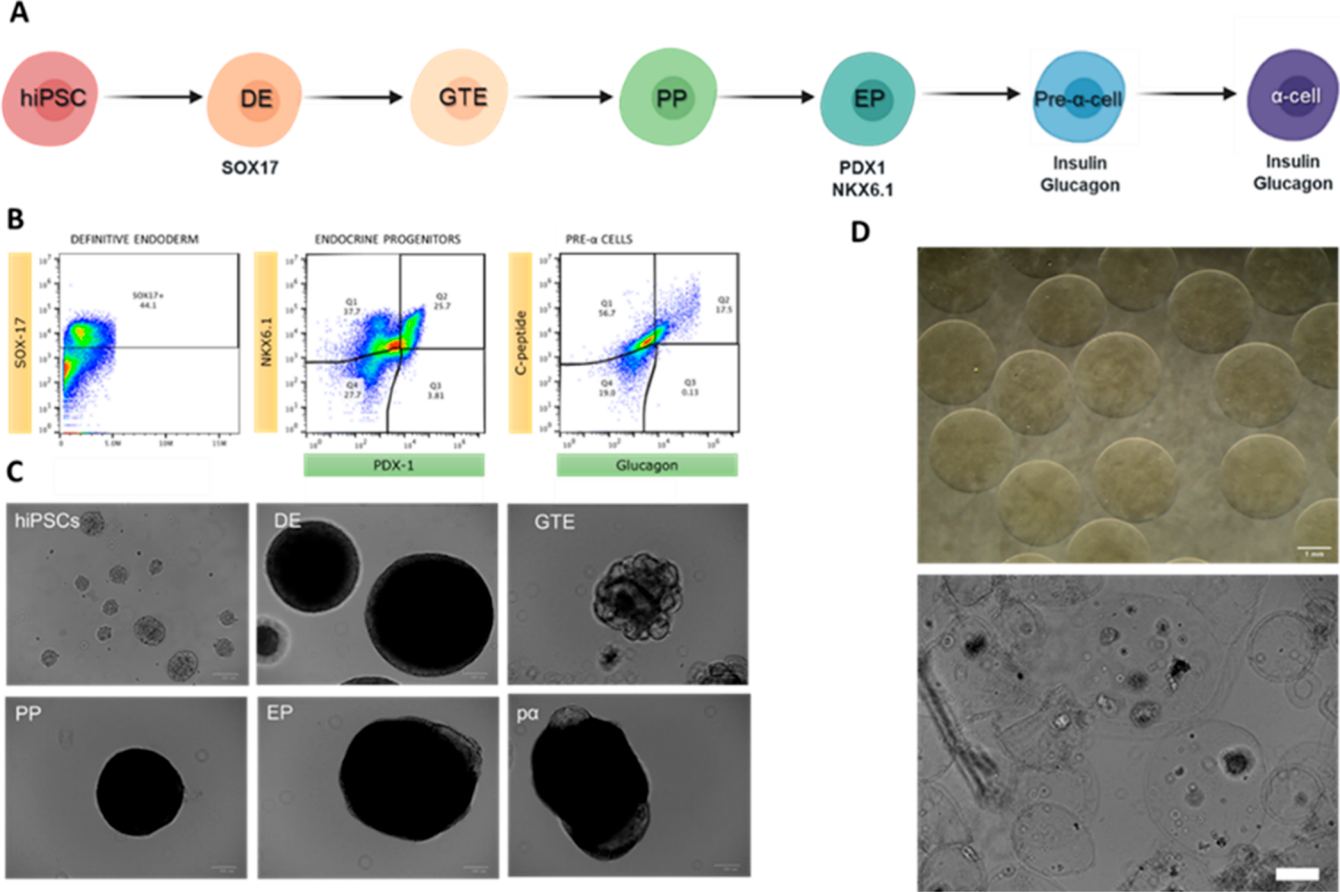
α-Cell differentiation from WLS-4D1 hiPS cell line. (A) Schematic representation of the differentiation strategy for the stepwise generation of pancreatic α-cells. Legend: hiPSC: induced pluripotent stem cell; DE: definitive endoderm; GTE: gut tube endoderm; PP: pancreatic progenitors; EP: endocrine progenitors; and pα: pre-α-cells. (B) Flow cytometry dot plots of SOX17 expression in definitive endoderm cells, NKX6.1 and PDX-1 expression in endocrine progenitors cells, and C-peptide and glucagon expression in pre-α-cells stage. (C) Representative brightfield images of the morphology of WLS-4D1 cells during differentiation until pre-α-cells. Scale bar: 100 μm. (D) Representative stereoscopic images of EXN-loaded pH-sensitive NPs encapsulated within alginate microspheres (upper image); representative bright-field image of differentiated cells encapsulated within the alginate system (lower image). Scale bar: 100 μm.

The same cell line (WLS-4D1) was induced toward pre-α-cells following a different stepwise protocol (Figure 5A,C) in static suspension culture,^33^ instead of adherent conditions. For that, WLS-4D1 cells were successfully adapted to suspension conditions before they proceeded to differentiation experiments. In order to assess their quality before undergoing differentiation, hiPSCs were stained for key pluripotency markers (OCT-4, SSEA-4, and TRA-1–81) and a differentiation marker (SSEA-1), as a negative control. Expression of OCT4, SSEA-4, and TRA-1–81 and the absence of SSEA-1 expression (Figure S2) confirmed the pluripotency of cultured cells. These results hold significant importance for further differentiation experiments since differentiation efficiency relies on the quality of the initial hiPSCs.

The differentiation was followed by assessing the expression of specific markers by flow cytometry at day 4 (end of DE stage), day 13 [end of endocrine progenitors (EP) stage], and day 20 [end of pre-α-cells (pα) stage] (Figure 5B). At the end of DE, SOX17 expression was approximately 44%, which was slightly lower than previously reported by other authors.^26^ The low expression of SOX17 is associated with a lower commitment to the endoderm lineage, which could compromise the efficiency of the differentiation protocol.^26^ On day 13 (end of EP stage), the expression of PDX1 and NKX6.1 was evaluated, and around 4% of the cell population was PDX1^+^/NKX6.1^–^, while 26% was PDX1^+^/NKX6.1^+^. At this stage in α-cell differentiation, the desired cell population is PDX1^+^/NKX6.1^–^^34^ since PDX1 expression directs differentiation into endocrine progenitors and the absence of NKX6.1 leads cells toward the α-cell phenotype and expression of glucagon.^27,33^ At the end of the pα stage (day 20), glucagon (GLU) and C-peptide (C-pep) expressions were assessed, and 17.5% of the differentiated cells were C-pep^+^/GLU^+^, which was similar to previously reported differentiation efficiencies at this stage.^33^ Once transplanted, these cells can convert to mature α-cells in vivo.^33^ Both SC-β-cells and SC-pre-α-cells were encapsulated within alginate microspheres (2% w/v) as well as the glucose-responsive NPs (as shown in Figure 5D) to build the system that could be tested in further studies.

### In Vivo Study in Diabetic Mice

**Figure 6:**
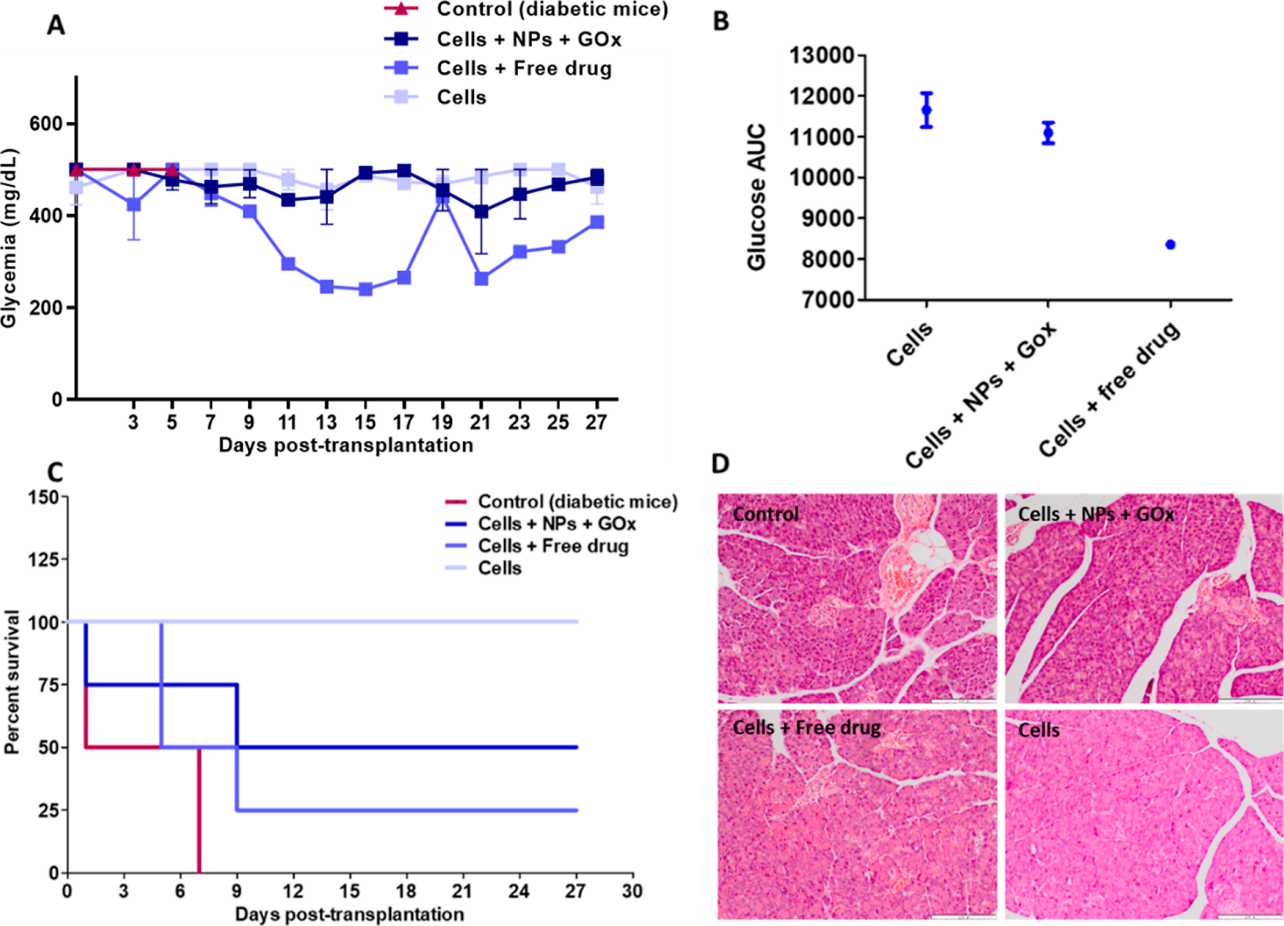
In vivo characterization of β- and pre-α-cells immobilized in an alginate matrix with glucose-responsive NPs. (A) Blood glucose concentration (mg/dL) and (B) AUC values for blood glucose levels of mice during the 27 days after transplantation. Cells were encapsulated either alone (Cells), in combination with EXN-loaded glucose-responsive NPs and glucose oxidase (Cells + NPs + GOx), or in combination with EXN freely dispersed in the alginate matrix (Cells + Free drug). Blood glucose levels were monitored every 2 days for 27 days. Values are represented as mean ± SEM (A) and mean ± SD (B). (C) Kaplan–Meier survival curve for mice transplanted with the different treatments. (D) Representative histology images of the pancreata of streptozocin-induced diabetic mice. Pancreatic sections of animals from different groups were stained with H&E: diabetic mice without treatment (Control); diabetic animal transplanted with Cells + NPs + GOx; diabetic animal transplanted with Cells + free EXN; and diabetic animal transplanted with Cells alone. Scale bar: 200 μm.

In an exploratory in vivo experiment, SC-β-cells and SC-pre-α-cells were encapsulated within alginate microspheres (2% w/v), either alone (Cells), with the GLP-1 analogue EXN freely dispersed (100 nM) in the alginate matrix (Cells + Free drug), or with EXN-loaded glucose-responsive NPs (corresponding to 100 nM drug) and the enzyme glucose-oxidase (10 μg/mL) (Cells + NPs + GOx). SC-β- and pre-α-cells were combined in the alginate microspheres following a ratio of 60:40 (β-cells: pre-α-cells) to roughly recapitulate the proportion found in primary human islets. Alginate microspheres were then transplanted into the peritoneal cavity of single high-dose streptozotocin-induced diabetic C57BL/6 male mice (Figure S3). Animals were considered diabetic when at least 2 consecutive nonfasting blood glucose readings were ≥400 mg/dL. Animals were followed throughout 27 days regarding weight (Figure S4) and blood glucose levels (Figure 6A). The transplantation of differentiated cells embedded in an alginate matrix, regardless of the presence of EXN-loaded NPs or free drug, led to an improvement in the survival of transplanted animals since the controls (diabetic animals without treatment) had a higher mortality percentage, with 100% at day 7 after transplantation (Figure 6C). This was also a consequence of the severity of the induced disease that, in the absence of treatment, led to animals’ death due to β-cells destruction. To confirm this, the pancreata of animals were collected and treated with hematoxylin and eosin (H&E) (Figure 6D).

Animals from the different groups showed an altered pancreatic structure in both the exocrine and endocrine pancreas. The acinar cells presented some hypertrophy and vacuolization, while Langerhans islets were nearly absent or presented reduced dimensions, confirming the success of diabetes induction and maintenance.

The coencapsulation of differentiated cells with EXN-loaded NPs appears to result in a slight reduction in hyperglycemia in diabetic mice when compared to encapsulated cells alone (Figure 6A). This trend is supported by the lower area under the curve (AUC) values obtained for this group (Figure 6, part B). Although this effect is more pronounced when the cells are encapsulated with the drug freely dispersed in the alginate matrix (Cells + Free drug), the higher mortality rate of this group jeopardizes any conclusion. The duration of the in vivo experience (27 days) may have impaired the observation of a clear reduction in the blood glucose levels of animals since it typically takes approximately 2 weeks after cell transplantation to start to observe a reduction in the blood glucose values and around 40 days to achieve normoglycemic levels.^31,32^ Moreover, the low differentiation efficiencies and the presence of polyhormonal β-cells in the final stage of differentiation may have also diminished the efficacy of the developed system since the insulin release capacity of SC-β-cells was compromised in vitro. The number of cells transplanted per animal (∼1.7 million) may have also limited the impact on blood glucose levels since the majority of published studies use between 2 and 5 million cells per animal, taking into account the marker expression and depending on the implantation site.^31,32,35−37^

Regarding the release of EXN from NPs, it is anticipated that during the initial 48 h after transplantation, the peptide will experience an initial burst release, followed by a slower release, as evidenced by the in vitro results (Figure 1B). Additionally, it is expected that an increase of up to 30% in EXN release following meals will occur, given that the rise in glucose levels transiently decreases the pH in the microenvironment of the transplanted system, thereby accelerating the NPs solubilization. Although it would be desirable to determine how long this formulation lasts until NPs are depleted, it presents significant challenges in both in vitro and in vivo settings. Specifically, the rate of drug release within the transplanted system and consequently the depletion rate of the drug content from NPs are significantly influenced by glycemic levels. These levels fluctuate throughout the day in response to the animal’s activity, metabolism, and health status, and replicating these dynamic variations in vitro poses substantial difficulties, in addition to the inherent limitations of in vitro systems. Conversely, the determination of this specific parameter in vivo would require the use of a considerable number of animals, given the necessity of conducting several experimental end points. Given the scope of this study and the principles of the 3Rs, the authors considered it more relevant to assess other parameters such as glycemia and survival rates after transplantation.

Nevertheless, considering that transplanted cells experience several acute stressors immediately after transplant (including hypoxia, nutrient deprivation, and inflammation)^38^ and that EXN promotes β-cells proliferation and differentiation,^23−25^ its release is especially important in the initial phase after transplantation to reduce the degree of β-cell loss. For these reasons, the initial EXN burst release observed in vitro (Figure 1B) may be advantageous in improving the survival of the transplanted SC-β-cells immediately after transplantation, followed by a slow release in an intermediate phase in a glucose-dependent manner to promote insulin secretion. In later stages, after the depletion of the drug content from the NPs, the transplanted cells should be able to independently survive and function in their environment.

Previous studies have shown that hESC/iPSC-derived pancreatic progenitor cells when transplanted into ectopic sites in immunodeficient or T1D mice can undergo further differentiation and maturation in vivo.^27,39,40^ In these studies, PP cells differentiated into glucose-responsive insulin-secreting cells in vivo, which were able to reverse diabetes in recipient mice. This shows that some growth factors and cues that are present in vivo are still missing in fully in vitro differentiation protocols. However, while it can be advantageous to transplant PP cells and allow the last differentiation phases to occur in vivo, there are some tumorigenicity and safety risks associated.^9^ Alternatively, to SC-β-cells differentiated until stage 7, as in the present study, stage 6 SC-β-cells could be incorporated into the system described here and allowed to complete their maturation in vivo. This would be an interesting avenue for further investigation, as other authors have reported that the maturation of stage 6 SC-β-cells in vivo depends on the transplantation site and that these cells did not lead to overgrowths or tumor formation after transplantation.^41^ By combining these cells with EXN-loaded NPs, their initial release after transplant could play an important role in further stimulating stage 6 SC-β-cell differentiation and maturation in vivo.

Considering future clinical applications, an almost totally in vitro differentiation protocol that originates from fully functional and mature β- and α-cells will be desirable. Despite differentiation protocols have been improved and optimized in the past few years, this is not yet the reality. Therefore, the integration of advancements in different areas such as encapsulation techniques, differentiation protocols, and gene editing strategies aimed at immune system evasion will facilitate the development of a cell replacement therapy for T1D.

## Conclusions

In this study, GLP-1 analogue-loaded NPs were successfully developed, showing a pH-dependent release profile. These NPs were later coencapsulated in alginate microspheres with glucose oxidase and SC-pancreatic cells. Microspheres were then transplanted into diabetic mice, and their glycemic levels were monitored for 1 month, as well as their survival rates. Despite the limitations of the present study, there was a clear improvement in the survival of the animals after the transplantation of either the complete system (Cells + NPs + GOx), Cells + EXN, or only cells when compared to the control group. Besides, diabetic mice transplanted with Cells + EXN showed a decrease in glucose levels. Additional research is needed to further evaluate the impact of this cell therapy in the STZ-induced diabetic mouse model, for instance, by increasing the number of animals per group and also the duration of the study. Nevertheless, we expect this work to provide a starting point for the development of novel cellular therapies for T1D that explore the synergy of nanomedicines and stem cell-based approaches.

## Materials and Methods

### Materials

Poly(lactide-*co*-glycolide) (PLGA) polymer (PURASORB PDLG 7502A 75/25 dl-lactide/glycolide copolymer 0.2 dL/g acid terminated) was kindly offered by Corbion (The Netherlands), Eudragit 100 was a kind offer from Evonik (Germany), and SMG was kindly offered by Novo Nordisk (Denmark). Ethyl acetate and amicon Ultra-15 centrifugal filter units (molecular weight cutoff: 100 kDa) were acquired from Merck Millipore (U.S.A.), EXN from Advanced ChemBlocks Inc. (U.S.A.), Kolliphor P 407 from BASF (Germany), dimethylformamide (DMF) from Honeywell (U.S.A.), trifluoroacetic acid (TFA) from Acros Organics (Belgium), and acetonitrile (ACN) was acquired from Fisher Scientific (U.S.A.).

### Nanoparticle Preparation

GLP-1 analogue-loaded NPs were developed using a modified solvent emulsification-evaporation method based on a water-in-oil-in-water (w/o/w) double emulsion technique, previously described by our group.^16^ Briefly, the organic phase consisted of a polymer solution at 40 mg/mL. In the final formulation, this solution was composed of 50% PLGA (MW = 17 kDa) and 50% Eudragit E100 (MW = 47 kDa) dissolved in ethyl acetate overnight at room temperature (RT). The drug (either EXN or SMG) was dissolved in Milli-Q water at a concentration of 44 mg/mL of which 100 μL was added to 1 mL of the organic phase. Afterward, this mixture was homogenized for 30 s with 70% amplitude, using a Vibra-Cell (VC 50) Ultrasonic Processor from Sonics & Materials, Inc. (U.S.A.) with a 2 mm sonication probe, to create the first emulsion water-in-oil (w/o). Then, the first emulsion was transferred to a tube containing 4 mL of a Kolliphor P 407 aqueous solution (1% w/v) and sonicated for 15 s under the same conditions. The second emulsion was immediately transferred to 15 mL of a Kolliphor P 407 aqueous solution (1% w/v) and kept under magnetic stirring at 300 rpm (revolutions per minute) for 3 h to promote ethyl acetate evaporation. NPs were washed three times with Milli-Q water to remove nonencapsulated drug and recovered by ultrafiltration using Amicon Ultra-15 centrifugal filter units (100 kDa cutoff). Unloaded NPs were produced following the same protocol without the addition of peptides.

### Particle Size and Zeta Potential Analysis

NPs were characterized regarding their average size and PDI by DLS and zeta potential through laser Doppler electrophoresis using a Zetasizer Nano ZS (Malvern Panalytical Ltd., UK). To perform these measurements, samples were diluted (1:100) in a 10 mM sodium chloride aqueous solution. The values reported are the average of three separate measurements.

### Morphological Evaluation

The morphological features of NPs were analyzed by scanning electron microscopy (SEM) using a high-resolution (Schottky) environmental scanning electron microscope with X-ray microanalysis and electron backscattered diffraction analysis: FEI Quanta 400 FEG ESEM (EDAX Genesis X4M). Liquid NPs were mounted over a silicon wafer support and coated with an Au/Pd thin film by sputtering for 40 s with a 15 mA current using the SPI Module Sputter Coater equipment.

### Freeze Drying of Nanoparticles

After washing NPs, these were resuspended in 2 mL of Milli-Q water and poured into semistoppered glass vials with slotted rubber closures for lyophilization. Samples were frozen at −80 °C for 12 h, followed by lyophilization using a FreeZone 2.5 Plus from Labconco (U.S.A.) at 0.09 mbar for 72 h at −80 ± 5 °C.

### Drug Association Efficiency and Loading Degree

The amount of loaded drug was directly quantified by high-performance liquid chromatography (HPLC) to determine the AE and DL of the developed formulations by dissolving 3 mg of freeze-dried NPs in 5 mL of DMF. AE and DL were determined following eqs 1 and 2, respectively.
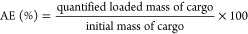
1
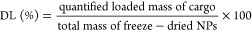
2

EXN or SMG were quantified by reversed-phase HPLC with fluorescence detection using a Shimadzu UFLC Prominence System equipped with two pumps LC-20AD, an autosampler SIL-20AC, a column oven CTO-20AC, a degasser DGU-20A5, a fluorescence detector RF-10AXL, a system controller CBM-20A, and the software LC solution, version 1.24 SP1.

For SMG quantification, a Synergi Max-RP C12 column (4 μm; 4.6 mm × 250 mm) (Phenomenex, Inc., U.S.A.) was used as a stationary phase with a LiChrospher 100 RP-18 (5 μm) LiChroCART 4–4 guard column (Merck, U.S.A.). Chromatographic analysis was performed by using a gradient method at a flow rate of 1 mL/min. Mobile phases consisted of 0.1% TFA in ultrapure water (eluent A) and 0.1% TFA in acetonitrile (eluent B). The gradient started at 60% eluent A, decreasing to 20% during the first 10 min. Then, eluent A increased again to 60% from 10 to 11 min and was kept constant from 11 to 18 min. The injection volume was 20 μL, and detection was performed by fluorescence, with excitation at 295 nm and emission at 350 nm.

For EXN quantification, a LiChrospher 100 RP-18 (5 μm) LiChroCART 125-4 column was used as the stationary phase with a LiChrospher 100 RP-18 (5 μm) LiChroCART 4-4 guard column (both from Merck, U.S.A.). Chromatographic analysis was performed by using a gradient method at a flow rate of 0.9 mL/min. Mobile phases consisted of 0.1% TFA in ultrapure water (eluent A) and acetonitrile (eluent B). The gradient started at 67% of eluent A decreasing to 55% during the first 10 min. Then, eluent A increased again to 67% from 10 to 10.1 min and remained constant until 24 min. The column was maintained at 35 °C during the analysis, and the injection volume was 20 μL. Detection was performed by fluorescence with excitation at 295 nm and emission at 355 nm.

### Peptide In Vitro Release Studies

Peptide release in vitro studies were performed after washing NPs to eliminate the nonencapsulated drug. For the in vitro release study, the “sample and separate” method^42^ was used. Peptide-loaded NPs were dispersed in 5.0 mL of 25 mM phosphate buffer (pH 7.4) or 25 mM acetate buffer (pH 5) and incubated at 37 °C under orbital agitation at 100 rpm. Each condition was analyzed in triplicate. Aliquots of 200 μL were collected at specific time points (5 min, 30 min, 1, 2, 4, 8, 24, and 48 h), and the volume was replaced with the respective buffer, kept at the same temperature. Each aliquot was centrifuged at 10,000*g* for 10 min, and the supernatant was stored at −20 °C until further analysis by HPLC, as previously described.

### Circular Dichroism Spectroscopy

In order to evaluate the secondary structures of EXN and SMG released from NPs at different time points, CD spectroscopy was performed. Measurements were conducted using a Jasco J815 CD spectrophotometer (Jasco Incorporated, U.S.A.), and the lamp housing was purged with nitrogen. Spectra were obtained from 190 to 260 nm using a 0.1 cm cell, a bandwidth of 1 nm, a data pitch of 1 nm, a digital integration time (D.I.T.) of 2 s, a scanning speed of 50 nm/min, and an accumulation of 6 scans per sample. Since the voltage was higher than 600 V and absorbance was also high at wavelengths below 198 nm, data are represented between 198 and 260 nm. CD samples were obtained at specified time points from the in vitro release studies. Freshly prepared solutions of each peptide at 200 μg/mL were used as a reference (native peptide). CD raw data were converted to mean residue ellipticity at wavelength λ ([θ]_mrw,λ_) given by
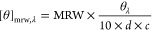
3where MRW is the mean residue weight, θ_λ_ is the observed ellipticity (degrees) at wavelength λ, *d* is the path length (cm), and *c* is the concentration (g/mL). The MRW for the peptide bond is calculated by MRW = *M*/(*N* – 1), where *M* is the molecular mass of the peptide (in Da), and *N* is the number of amino acids (the number of peptide bonds is *N* – 1).^43^

### Cell Culture Conditions

INS-1E cells were used between passages 80 and 90 and were routinely cultured in RPMI 1640 medium containing 10 mM HEPES (Merck, U.S.A.), supplemented with 10% V/V of fetal bovine serum (FBS), 1× Penicillin–Streptomycin Solution (100 IU/mL Penicillin and 100 μg/mL Streptomycin) (Corning, U.S.A.), and 50 μM of β-mercaptoethanol. INS-1E cells were cultured in 75 cm^2^ T-flasks at 37 °C, 5% CO_2_, and 95% relative humidity with medium changes every 2 or 3 days.

### hiPSC Source

WLS-4D1 hiPSC cell line was developed and kindly provided by the lab of William Stanford (Ottawa Hospital Research Institute).

### Adherent Culture of hiPSCs

Regular adherent culture and maintenance of WLS-4D1 cells were performed on tissue culture-treated 6-well plates (Falcon, U.S.A.) coated with Matrigel (Corning, U.S.A.). After thawing, Matrigel was diluted in cold DMEM/F-12 with 15 mM HEPES buffer (STEMCELL Technologies, Canada), according to the manufacturer’s instructions. Diluted Matrigel solution was immediately used for coating tissue culture plates using 1 mL/well. Coated plates were incubated at room temperature for at least 1 h, and excess Matrigel solution was removed from the wells immediately before use.

WLS-4D1 cells were maintained in mTeSR Plus (STEMCELL Technologies, Canada) with daily change in medium changes. Cells were passaged as small aggregates (50–200 μm) every 5–7 days, depending on the aggregate density. Passaging was performed using ReLeSR (STEMCELL Technologies, Canada), an enzyme-free passaging reagent, according to the manufacturer’s instructions. The well to be passaged was washed with D-PBS (Dulbecco’s phosphate-buffered saline) without Ca^++^ and Mg^++^ (STEMCELL Technologies, Canada) and incubated with ReLeSR at room temperature for 1 min. Afterward, ReLeSR was removed, leaving only a thin layer of liquid covering the colonies. After incubation at 37 °C for 4–5 min, mTeSR Plus was added. To detach the aggregates, the plate was firmly tapped on the side for approximately 30 s. Aggregates were transferred to a tube containing mTeSR Plus, diluted at the desired concentration, and reseeded in a Matrigel-coated 6-well plate. Cells were incubated at 37 °C and 5% CO_2_ and undisturbed for 24 h.

Gentle Cell Dissociation Reagent (STEMCELL Technologies, Canada) was used for the preparation of single-cell suspensions. After washing with D-PBS without Ca^2+^ and Mg^2+^, cells were incubated with Gentle Cell Dissociation Reagent for 10 min at 37 °C, resuspended, and transferred to a tube containing an equal volume of DMEM/F-12. Cells were then centrifuged at 300*g* at 20 °C for 5 min and resuspended in mTeSR Plus supplemented with 10 μM Y-27632 (STEMCELL Technologies, Canada).

### Suspension Culture of hiPSCs

To start a suspension culture from an adherent culture, WLS-4D1 was dissociated into single cells at the time of confluency. The obtained single-cell suspension was diluted in mTeSR Plus supplemented with 10 μM Y-27632 (STEMCELL Technologies, Canada) to an optimal seeding density of ∼2 × 10^5^ cells/mL (static suspension culture) or ∼3 × 10^5^ cells/mL (dynamic suspension culture). The cell suspension was transferred to a T-flask (SPL Life Sciences, Korea) and incubated in the upright position at 37 °C with 5% CO_2_ for static suspension culture. Alternatively, for dynamic suspension culture, the cell suspension was transferred to a 25 mL spinner-flask (Bellco Glass, U.S.A.) and incubated at 37 °C with 5% CO_2_ in a magnetic stirrer (Variomag Biosystem 4, Thermo Fisher Scientific, U.S.A.) with a rotation speed of 5 rpm. After 48 h, the medium was replaced by mTeSR Plus without Y-27632. Afterward, medium change was performed daily.

Suspension cultures were passaged every 4–5 days. For passaging, the suspension culture was collected into a tube and centrifuged at 300*g* at 20 °C for 5 min. The aggregates were then dissociated into single cells using Gentle Cell Dissociation Reagent and seeded in mTeSR Plus supplemented with 10 μM Y-27632 at a concentration of ∼2 × 10^5^ cells/mL (static suspension culture) or ∼3 × 10^5^ cells/mL (dynamic suspension culture).

For reseeding in adherent culture, 200–300 aggregates were used per well of a 6-well plate. The required suspension volume was collected and centrifuged at 300*g* at 20 °C for 5 min. The aggregate pellet was resuspended in mTeSR Plus supplemented with 10 μM Y-27632, and 2 mL of the aggregate suspension was seeded per well. On the following day, the medium was replaced by mTeSR Plus without Y-27632, and the culture was maintained as a regular adherent culture.

### hiPSC Differentiation into Pancreatic β-Cells

Differentiation of WLS-4D1 into pancreatic progenitors (stage 4) was performed using STEMdiff Pancreatic Progenitor Kit (STEMCELL Technologies, Canada), according to the manufacturer’s instructions. On day 14 of the differentiation (end of stage 4), hiPSC-derived pancreatic progenitors were transferred to an air–liquid interface to further differentiate into mature β-cells, following the protocol proposed by Rezania et al.^31^

Briefly, hiPSCs were seeded as single cells in Matrigel-coated 6-well plates at a density of 2.5 × 10^6^ cells/well in order to reach 90–100% confluency on the following day. When stage 4 of differentiation was reached, cells were treated for 4 h with MCDB 131 medium (Gibco, U.S.A.) supplemented with 10 μM Y-27632. Afterward, cells were dissociated into single cells using Gentle Cell Dissociation Reagent and centrifuged at 300*g* for 5 min at 20 °C. The obtained cell pellet was resuspended in MCDB 131 medium supplemented with 10 μM Y-27632 at a density of 0.5 × 10^5^ cells/μL. On the apical side of 6-well plate filter inserts (Falcon, U.S.A.), 5–10 μL of the cell suspension was spotted (0.25–0.5 × 10^6^ cells/spot) with a total of around 10 spots per well. A volume of 1.5 mL of medium was used per well, and the mixture was added to the basolateral side of each insert. The differentiation protocol required daily medium changes, and further details can be found in the Supporting Information Methods.

### hiPSC Differentiation into Pancreatic α-Cells

Differentiation of hiPSCs into pancreatic pre-α-cells was performed following the protocol proposed by Peterson et al.^33^ WLS-4D1 hiPSCs were seeded as single cells in a suspension culture at a concentration of 2 × 10^5^ cells/mL in a T-75 flask in the upright position (static suspension). Cells were incubated at 37 °C with 5% CO_2_, and differentiation was initiated 2 days after seeding, when aggregates presented an average size of 100–200 μm. Further details regarding medium composition and medium changes can be found in the Supporting Information Methods.

### Flow Cytometry

Cells were analyzed by flow cytometry at several time points of the differentiation protocols. Cells were dissociated into single cells using Gentle Cell Dissociation Reagent, resuspended in mTeSR Plus supplemented with 10 μM y-27632, and counted using a hemocytometer. For staining of intracellular antigens, 1 × 10^5^ – 1 × 10^6^ cells were aliquoted per sample into a 5 mL round-bottom tube. Cells were centrifuged at 300*g* at 4 °C for 5 min, resuspended in 100 μL of diluted Zombie Green Fixable Viability Kit (BioLegend, U.S.A) solution (1:1000 in PBS), and incubated on ice in the dark for 15 min (optional step, performed only for differentiation into α-cells). After incubation, 2 mL of 2% *V*/*V* FBS in PBS solution was added per tube, and the samples were centrifuged at 300*g* at 4 °C for 5 min. Cells were fixed using 2% PFA for 15 min on ice. Afterward, 1 mL of 2% *V*/*V* FBS in PBS solution was added per tube, and the samples were centrifuged at 300*g* at 4 °C for 5 min. Cells were then resuspended in 500 μL of saponin permeabilization buffer (SPB), which consisted of 1 mg/mL saponin (Sigma-Aldrich, U.S.A.) and 1% w/v BSA in D-PBS without Ca^++^ and Mg^++^, and incubated at room temperature for 15 min. After centrifugation at 300*g* at 4 °C for 5 min, cells were resuspended in the antibody mix (or SPB for the unstained control) using 100 μL per sample. The antibodies used as well as respective dilutions and incubation conditions are listed in Table S1. After incubation, 1 mL of SPB was added to each tube and samples were centrifuged at 300*g* at 4 °C for 5 min. Cells were resuspended in 2% *V*/*V* FBS in PBS and filtered using a 100 μm filtration nylon membrane. Samples were placed on ice and analyzed using the BD Accuri C6 flow cytometer (BD Biosciences, U.S.A). FlowJo version 10 software was used to process flow cytometry data.

### Cell Encapsulation

For alginate sterilization, 1% w/v low-viscosity alginate (PRONOVA UP LVG sodium alginate, NovaMatrix, Norway) solution was prepared in sterile 0.9% w/v NaCl (Honeywell, U.S.A) and filtered using a Steriflip filter unit (Merck Millipore, U.S.A). The sterile solution was lyophilized for 72 h and stored at −20 °C until use.

For encapsulation, presterilized alginate powder was dissolved at the desired concentration in sterile 0.9% w/v NaCl and stirred overnight at 4 °C.

Cells cultured in suspension were encapsulated as aggregates, while cells cultured in adherent conditions were encapsulated as single cells. Aggregate/cell suspension was centrifuged at 200*g* at 20 °C for 5 min, and the pellet was resuspended in sterile alginate solution. The aggregate/cell suspension in alginate was loaded into a 1 mL syringe (Henke Sass Wolf, Germany). Extrusion was performed under a coaxial air flow using a Var J1 encapsulation unit (Nisco Engineering AG, Switzerland) and a syringe pump, as previously described.^44^

Sterile 100 mM CaCl_2_ (VWR, U.S.A.) solution in 0.9% w/v NaCl was used as the cross-linking solution. After extrusion, microspheres were allowed to cross-link for 10 min, washed with DMEM/F-12, and transferred to a 6-well plate. Microspheres were observed using a ZOE fluorescent cell imager and an inverted microscope.

Encapsulated cells were maintained in their respective medium. In the case of the encapsulation of hiPSCs as single cells, the culture medium was supplemented with 10 μM Y-27632 during the first 24 h after encapsulation.

### Glucose-Stimulated Insulin Secretion

In order to assess the function of stem cell-derived β-cells (SC-β-cells) (stage 7 of differentiation) and the glucoregulatory effect of EXN and SMG on INS-1E cells, a GSIS assay was performed, as described by Hogrebe et al.^45^ INS-1E cells were seeded at 2 × 10^5^ cells/mL in 24-well plates and cultured for 5 days, refreshing media on the third day. Cells were washed three times with Krebs buffer (Krb) and then preincubated in Krb for 30 min to remove residual insulin. Afterward, cells were washed once, incubated in low (2 mM) glucose Krb for 30 min (at 37 °C and 5% CO_2_), and supernatants were collected. Then, cells were incubated in high glucose (20 mM) Krb for 30 min, and the supernatant was collected. Krb buffer in low and high incubation steps had different concentrations of EXN or SMG. Finally, cells were incubated in Krb containing 2 mM glucose and 30 mM KCl for 30 min, and then the supernatant was collected. KCl-triggered depolarization increases cytosolic Ca^2+^ levels, leading to a peak in insulin secretion.

Supernatant samples containing secreted insulin were stored at −80 °C until further quantification. At the end of the experiment, cells were detached using trypsin/EDTA (Sigma-Aldrich, U.S.A.), and the cell number was counted manually using trypan blue and a hemocytometer.

Afterward, cells were lysed to assess intracellular insulin content. Cells were centrifuged at 300*g* for 5 min at 4 °C and washed twice with cold PBS. Cells were then incubated with ice-cold lysis buffer [cOmplete protease inhibitor cocktail (Roche, Switzerland) + 1% triton X-100 (Thermo Fisher Scientific, U.S.A.) in PBS 1×] for 30 min on ice, followed by centrifugation at 2000*g* for 10 min at 4 °C. The supernatant was collected, and acid-ethanol extraction was performed to extract protein content from lysates. The lysate was mixed with acid-ethanol (EtOH) [0.18 M HCl in 96% (*V*/*V*) EtOH] in a 1:3 proportion of the lysate and acid-ethanol solution and incubated at 4 °C for 12 h. Samples were then centrifuged to separate cellular debris. The supernatant was stored at −80 °C until further analysis by enzyme-linked immunosorbent assay (ELISA).

The SI, which is extensively used as a quantitative descriptor for islet characterization, was determined by calculating the ratio between insulin secretion at high and low glucose.^46^

### Enzyme-Linked Immunosorbent Assay

Sandwich ELISA was used to quantify the proinsulin and insulin contents in GSIS samples. Briefly, high-binding 96-well plates (Corning Costar, U.S.A.) were coated with 50 μL of anti-insulin antibody (Abcam, ref ab8304) diluted 1:1000 in PBS and incubated overnight at 4 °C under orbital agitation at 65 rpm. After incubation, the wells were washed three times with 200 μL of 0.05% Tween-20 in PBS (PBST) and blocked with 100 μL of 4% skimmed milk (PanReac AppliChem, Spain) diluted in PBST for 1 h at RT. The wells were washed again three times with 200 μL of PBST. An insulin calibration curve was prepared in the range of 10 to 2000 pg/mL. Fifty microliters of standards and diluted samples were added in triplicates to wells and incubated for 2 h at RT. Wells were washed as described before, and 50 μL per well of HRP anti-insulin antibody (Abcam, ref ab28063), diluted 1:2000 in PBS, was added for 1 h at RT, followed by a washing step. Afterward, 100 μL per well of 1-Step Ultra TMB-ELISA Substrate Solution (Thermo Fisher Scientific, U.S.A.) was added and incubated in the dark for 15 min. Then, 100 μL of 2 M sulfuric acid was added to each well to stop the reaction. Absorbance was measured at 450 nm using a SynergyMx MultiMode microplate reader.

### In Vivo Study

Male C57BL/6 mice were acquired from Charles River Laboratories (Spain). Only male mice were used in this study since females are less sensitive to the toxin streptozotocin (STZ),^47^ which was used for T1D induction. All animals involved in the experiments were handled in accordance with good animal practice, as defined by the European Union Directive 2010/63/EU and the national Decreto-Lei n° 113/2013. All animal experimental procedures described in this work were approved by the AWERB (animal welfare and ethics review body) at *Instituto de Investigação e Inovação em Saúde* (i3S) and conducted at the i3S animal facility, under authorization by the *Direção-Geral da Alimentação e Veterinária* (DGAV).

Five animals were housed per cage and were allowed to acclimatize in the facility for at least 1 week. Mice were housed at the i3S animal facility with *ad libitum* access to water and standard rodent food, unless stated otherwise. Animals were kept under a 12 h light/dark cycle, with 45–65% humidity and temperatures between 20 and 24 °C. Mice were randomly assigned to each experimental group.

For the induction of T1D, 9-week-old mice were fasted for 4 h prior to STZ administration at 200 mg/kg by intraperitoneal injection. After STZ injection, 10% sucrose water (to reduce morbidity and mortality) and standard rodent food were provided. Mice were closely monitored every 2 h for 12 h for marked hypoactivity, unresponsiveness, or convulsions. Three days after STZ administration, 10% sucrose water was replaced by regular water. Eleven days after STZ administration, treatments were initiated (experimental day 0) by transplanting the alginate microspheres into the peritoneal cavity.

Before transplantation, buprenorphine (0.1 mg/kg) in a sterile saline solution was administered subcutaneously to ensure pain relief before the mice regained consciousness. Mice were then anesthetized with 5% isoflurane at 2.5 mL/min, which was reduced to 2% once the animal became unresponsive. Fur in the lower abdomen was shaved, and the skin was disinfected with chlorhexidine. An incision of 4–5 mm wide was made through the skin and muscle. Then the tip of the syringe loaded with the microspheres was slightly and gently introduced through the incision, and alginate beads were transplanted into the mouse (1.76 × 10^6^ cells/animal).

The incision was closed by suturing muscle and skin separately with absorbable suture wire (3–4 sutures). After surgery, animals received subcutaneous injections of buprenorphine (0.1 mg/kg) twice a day for 3 days. A schematic representation of the in vivo experiment and transplantation procedure is shown in Supporting Information Figure S3.

During the entire duration of the study, animals were carefully monitored regarding body weight, blood glucose levels, and their overall body condition. Blood glucose levels were measured with a FreeStyle Precision (Abbott, U.S.A.) glucose meter, using a sample of capillary blood collected by pricking the lateral tail vein with a 27G needle. Animals were not fasted before blood glucose measurements to avoid inducing more stress. However, those measurements were performed at approximately the same time of the day in order to minimize the influence of the students’ eating habits on the glucose levels measured. Animals were sacrificed on day 27 by CO_2_ asphyxiation, followed by cervical dislocation.

### Statistical Analysis

For statistical analysis, GraphPad Prism Software vs 9.0.0 (GraphPad Software Inc.) was used. Unless otherwise mentioned, statistical analysis was performed by using a one-way ANOVA followed by Tukey’s pairwise comparisons. The differences were considered to be significant at *p* < 0.05.

## Supporting Information Available

hiPSC differentiation into pancreatic β-cells and α-cells, immunocytochemistry, list of antibodies used for flow cytometry, NP in vitro release, quality control of pluripotency markers of hiPSCs before differentiation, schematic representation of the in vivo study and transplantation procedure, and animal weight variation (PDF)

## Abbreviations

AE: association efficiency
AUC: area under the curve
CD: circular dichroism
C-pep: C-peptide
DE: definitive endoderm
DL: drug loading
DLS: dynamic light scattering
DM: diabetes mellitus
EDS: energy dispersive spectroscopy
EP: endocrine progenitors
EXN: exenatide
E100: Eudragit 100
GLP-1: glucagon-like peptide 1
GLP-1R: glucagon-like peptide 1 receptor
GLU: glucagon
GOx: glucose oxidase
GSIS: glucose-stimulated insulin secretion
GTE: gut tube endoderm
hiPSCs: human induced pluripotent stem cells
MFI: mean fluorescence intensity
NKX6.1: Nirenberg and Kim homeobox 6.1
NPs: nanoparticles
PBS: phosphate-buffered saline
PDI: polydispersity index
PDX1: pancreatic and duodenal homeobox factor 1
PF: posterior foregut
PGT: primitive gut tube
PLGA: poly(lactic-*co*-glycolic acid)
PP: pancreatic progenitors
SC-β-cells: stem cell-derived β-cells
SEM: scanning electron microscopy
SI: stimulation index
SMG: semaglutide
SOX17: sex-determining region Y-box 17
STZ: streptozotocin
T1D: type 1 diabetes
ZP: zeta potential
