# Supplementary material for: GLP-1 Analogue-Loaded Glucose-Responsive Nanoparticles as Allies of Stem Cell Therapies for the Treatment of Type I Diabetes
Source: ACS Pharmacol Transl Sci. 2024 May 2;7(5):1650–63. doi: 10.1021/acsptsci.4c00173 (PMC11092009; doi:10.1021/acsptsci.4c00173)
Supplement: Supplementary file 1 — pt4c00173_si_001.pdf [file pt4c00173_si_001.pdf]

Supporting Information

GLP-1 analogue-loaded glucose-responsive nanoparticles as allies of stem cell therapies for the treatment of Type I Diabetes

Joana Moreira Marques [a,b,c], Rute Nunes [a,d], Ana Margarida Carvalho [a,b,e], Helena Florindo [f], Domingos Ferreira [c], Bruno Sarmento [a,b,d] *

[a] i3S - Instituto de Investigação e Inovação em Saúde, Universidade do Porto, Portugal.

[b] INEB - Instituto de Engenharia Biomédica, Universidade do Porto, Rua Alfredo Allen, 208, 4200-180 Porto, Portugal.

[c] UCIBIO - Applied Molecular Biosciences Unit, REQUIMTE, MedTech - Pharmaceutical Technology Laboratory, Drug Sciences Department, Faculty of Pharmacy, University of Porto, Portugal.

[d] IUCS-CESPU - Instituto Universitário de Ciências da Saúde, Gandra, Portugal.

[e] ICBAS - Instituto de Ciências Biomédicas Abel Salazar, Universidade do Porto, Rua de Jorge Viterbo Ferreira, 228, 4050-313 Porto, Portugal.

[f] Research Institute for Medicines (iMed.ULisboa), Faculty of Pharmacy, Universidade de Lisboa, Av. Prof. Gama Pinto, 1649-003 Lisbon, Portugal.

*Corresponding author:

Email: bruno.sarmento@i3s.up.pt

Supplementary Methods

hiPSCs differentiation into pancreatic β-cells

The differentiation protocol requires daily medium changes according to the following information (volumes are expressed per well of a 6-well plate):

• **Stage 1** (definitive endoderm)

Day 1: 1.96 mL Endoderm Basal Medium + 20 μL Supplement MR + 20 μL Supplement CJ.

Day 2: 1.98 mL Endoderm Basal Medium + 20 μL Supplement CJ.

• **Stage 2** (primitive gut tube)

Day 3: 1.96 mL Stage 2-4 Basal Medium + 20 μL Supplement 2A + 20 μL Supplement 2B.

Days 4 and 5: 1.98 mL Stage 2-4 Basal Medium + 20 μL Supplement 2B.

• **Stage 3** (posterior foregut)

Days 6, 7 and 8: 1.98 mL Stage 2-4 Basal Medium + 20 μL Supplement 3.

• **Stage 4** (pancreatic progenitors)

Days 9 – 13: 1.98 mL Stage 2-4 Basal Medium + 20 μL Supplement 4.

• **Stage 5** (endocrine progenitors)

Days 14 – 16: MCDB 131 medium supplemented with 20 mM glucose (Sigma-Aldrich, U.S.A.), 1.5 g/L sodium bicarbonate (Sigma-Aldrich, U.S.A.), 2 % *W*/*V* Bovine Serum Albumin, BSA (VWR, U.S.A.), 1x GlutaMAX™ (Gibco™, U.S.A.), 1:200 Insulin-Transferrin-Selenium-Ethanolamine, ITS-X, (Gibco™, U.S.A.), 0.25 μM SANT-1 (Merck Millipore, U.S.A.), 0.05 μM retinoic acid (Sigma-Aldrich, U.S.A.), 100 nM LDN193189 (Sigma-Aldrich, U.S.A.), 1 μM 3,3',5-Triiodo-L-thyronine sodium salt, T3, (Sigma-Aldrich, U.S.A.), 10 μM ALK5 inhibitor II (Enzo Life Sciences, U.S.A.), 10 μM zinc sulfate (Sigma-Aldrich, U.S.A.) and 10 μg/mL heparin (Sigma-Aldrich, U.S.A.).

• **Stage 6** (immature β-cells)

Days 17 – 24: CMRL medium (Gibco™, U.S.A.) supplemented with 20 mM glucose, 1.5 g/L sodium bicarbonate, 2 % *W*/*V* BSA, 1x GlutaMAX™, 1:200 ITS-X, 100 nM LDN193189, 1 μM T3, 10 μM ALK5 inhibitor II, 100 nM γ-secretase inhibitor XX (Merck Millipore, U.S.A.), 10 μM zinc sulfate and 10 μg/mL heparin.

• **Stage 7** (mature β-cells)

Days 25 – 31: CMRL medium supplemented with 20 mM glucose, 1.5 g/L sodium bicarbonate, 2 % *W*/*V* BSA, 1x GlutaMAX™, 1:200 ITS-X, 1 μM T3, 10 μM ALK5 inhibitor II, 1 mM N-acetyl cysteine (Sigma-Aldrich, U.S.A.), 10 μM Trolox (Merck Millipore, U.S.A.), 2 μM R428 (MedChemExpress, U.S.A.), 10 μM zinc sulfate and 10 μg/mL heparin.

hiPSCs differentiation into pancreatic α-cells

Medium changes were performed according to the following schedule. Aggregates were allowed to settle for ~5 – 10 min and the old medium was removed by aspiration.

• **Stage 1** (definitive endoderm)

Day 1: MCDB 131 medium supplemented with 8 mM glucose, 2.46 g/L sodium bicarbonate, 2 % *W*/*V* BSA, 2 mM GlutaMAX™, 1:50,000 ITS-X, 0.25 ascorbic acid (Sigma-Aldrich, U.S.A.), 100 ng/mL Activin A (R&D Systems, U.S.A.) and 3 μM CHIR99021 (Sigma-Aldrich, U.S.A.).

Day 2: MCDB 131 medium supplemented with 8 mM glucose, 2.46 g/L sodium bicarbonate, 2 % *W*/*V* BSA, 2 mM GlutaMAX™, 1:50,000 ITS-X, 0.25 ascorbic acid and 100 ng/mL Activin A.

• **<u>Stage 2</u>** (gut tube endoderm)

Day 4: MCDB 131 medium supplemented with 8 mM glucose, 1.23 g/L sodium bicarbonate, 2 % *W*/*V* BSA, 2 mM GlutaMAX™, 1:50,000 ITS-X, 0.25 ascorbic acid and 50 ng/mL FGF7 (STEMCELL™ Technologies, Canada).

• **<u>Stage 3</u>** (pancreatic progenitors)

Day 6: MCDB 131 medium supplemented with 8 mM glucose, 1.23 g/L sodium bicarbonate, 2 % *W*/*V* BSA, 2 mM GlutaMAX™, 1:200 ITS-X, 0.25 ascorbic acid and 2 μM retinoic acid.

Day 7: MCDB 131 medium supplemented with 8 mM glucose, 1.23 g/L sodium bicarbonate, 2 % *W*/*V* BSA, 2 mM GlutaMAX™, 1:200 ITS-X, 0.25 ascorbic acid, 2 μM retinoic acid and 200 nM LDN193189.

• **<u>Stage 4</u>** (endocrine progenitors)

Day 8: MCDB 131 medium supplemented with 8 mM glucose, 1.23 g/L sodium bicarbonate, 2 % *W*/*V* BSA, 2 mM GlutaMAX™, 1:200 ITS-X, 0.25 ascorbic acid and 200 nM LDN193189.

Days 9 and 11: MCDB 131 medium supplemented with 8 mM glucose, 1.23 g/L sodium bicarbonate, 2 % *W*/*V* BSA, 2 mM GlutaMAX™, 1:200 ITS-X and 0.25 ascorbic acid.

• **<u>Stage 5</u>** (pre-α-cells)

Days 13, 15, 17 and 19: MCDB 131 medium supplemented with 8 mM glucose, 1.23 g/L sodium bicarbonate, 2 % *W*/*V* BSA, 2 mM GlutaMAX™, 1:200 ITS-X, 0.25 ascorbic acid and 10 μM ALK5 inhibitor II.

Immunocytochemistry

Cultured cells were regularly assessed for pluripotency by immunostaining. At around 80 % confluency, cells were fixed with 4 % paraformaldehyde (PFA, Thermo Fisher Scientific, U.S.A.) in 1x PBS (phosphate-buffered solution) for 10 min at RT. For intracellular staining, fixed cells were permeabilized with 1 % *V*/*V* Triton™ X-100 (Fisher BioReagents™, U.S.A.) in 1x PBS for 5 min at RT. Fixed cells were incubated with blocking solution (BS) for 1 h at RT. BS composition was 1.5 % *W*/*V* BSA (Fischer BioReagents™, U.S.A.) and 5 % *V*/*V* FBS (Biochrom AG, Germany) in 1x PBS. Afterwards, cells were incubated with the primary antibody diluted in BS (1:100) overnight at 4 °C. The following primary antibodies were used: Anti-Human OCT4 (#60093), Anti-Human SSEA-4 (#60062), Anti-Mouse SSEA-1 (#60060) and Anti-Human TRA-1-81 (#60065). All primary antibodies were acquired from STEMCELL™ Technologies (Canada). After three washing steps with 1x PBS for 5 min, cells were incubated with the secondary antibody diluted in BS (1:500) for 1 h at RT in the dark. The following secondary antibody was used: F(ab')2-Goat anti-Mouse IgG (H+L) Cross-Adsorbed Secondary Antibody, Alexa Fluor™ 594 (#A-11020, Thermo Fisher Scientific, U.S.A.). Cells were washed 3 times with 1x PBS for 5 min and stained with 4′,6-diamidino-2-phenylindole dihydrochloride, DAPI, (#D9542, Sigma-Aldrich, U.S.A.) at 500 ng/mL for 5 min at RT. After one washing step with 1x PBS, 2 mL of 1x PBS were added to each well. Cell imaging was performed using ZOE™ Fluorescent Cell Imager (Bio-Rad, U.S.A.).

Supplementary Figures and Tables

Table S1. List of antibodies used for flow cytometry.

Antibody	Supplier	Reference	Dilution	Incubation conditions
APC-conjugated anti-human SOX17	R&D Systems (U.S.A.)	IC1924A	1:50	30 min, 4 °C
PE Mouse anti-PDX-1 (Clone 658A5)	BD Biosciences (U.S.A.)	562161	1:20	1 h, 2 – 8 °C
Alexa Fluor® 647 Mouse anti-NKX6.1 (Clone R11-560)	BD Biosciences (U.S.A.)	563338	1:20	1 h, 2 – 8 °C
PE Mouse Anti-NKX6.1 (Clone R11-560)	BD Biosciences (U.S.A.)	563023	1:40	30 min, 4 °C
Insulin (C27C9) Rabbit mAb (Alexa Fluor® 647 Conjugate)	Cell Signaling (U.S.A.)	#9008	1:80	1 h, 4 °C
Alexa Fluor® 647 Mouse Anti-C-Peptide (Clone U8-424)	BD Biosciences (U.S.A.)	565831	1:50	1 h, 4 °C
PE Mouse Anti-Glucagon (Clone U16-850)	BD Biosciences (U.S.A.)	565860	1:20	1 h, 4 °C

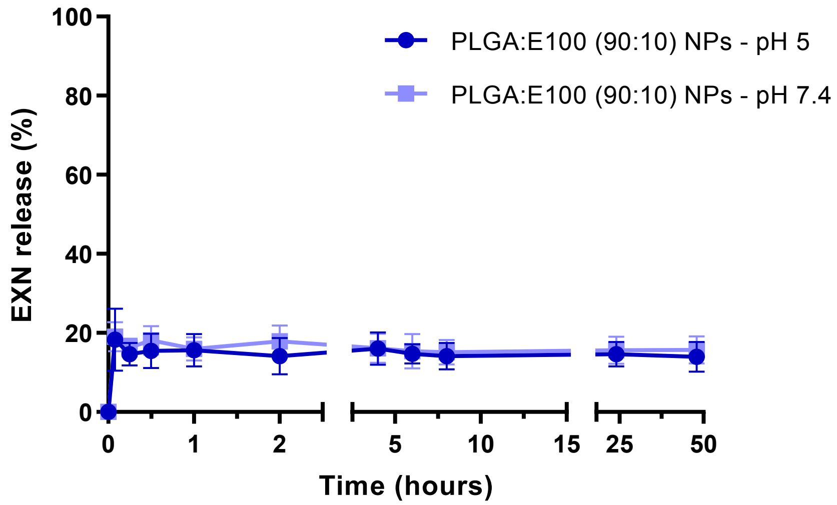

Figure S1. Release profile of EXN from PLGA:E100 (90:10) NPs at pH 5 and pH 7.4. Points and vertical bars stand for mean and standard deviation (SD), respectively.

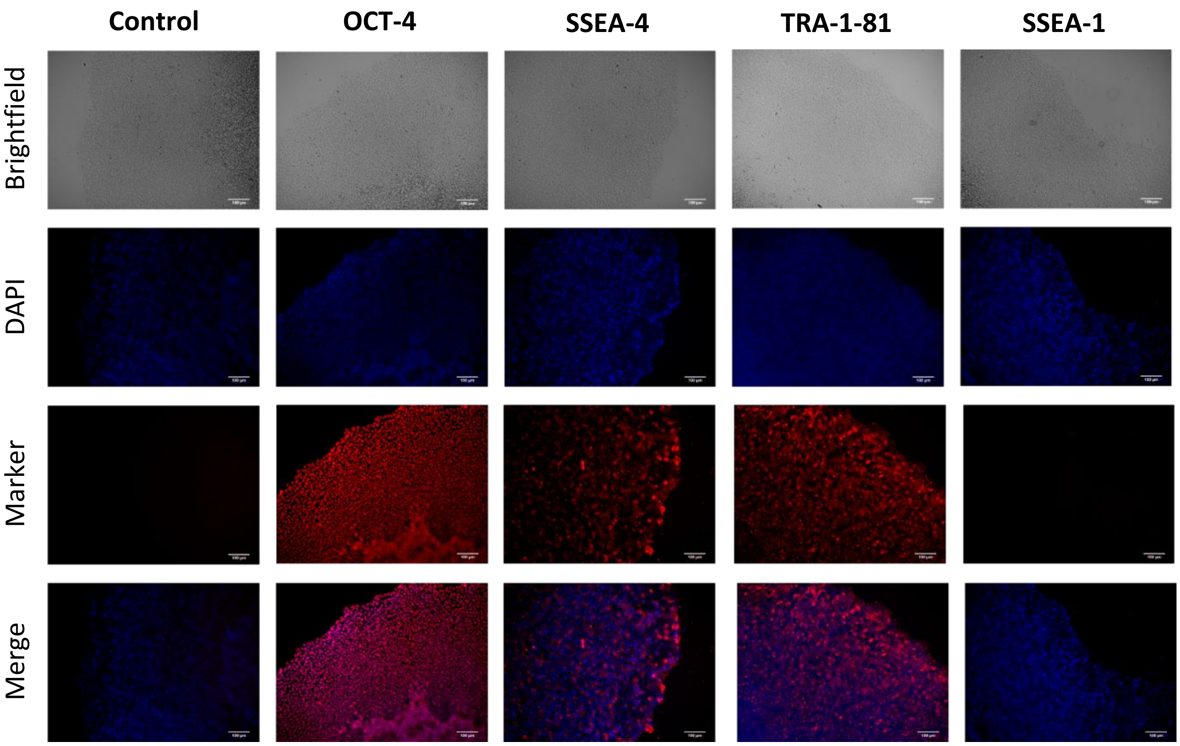

Figure S2. Quality control for pluripotency of hiPSCs previously cultured in static suspension conditions. WLS-4D1 cells were successfully maintained in static suspension culture for four passages and re-seeded in adherent culture two days after passage. Cells were stained for pluripotency markers (OCT-4, SSEA-4 and TRA-1-81) and differentiation marker (SSEA-1) in red and the nucleus (DAPI) in blue. Scale bar: 100 µm.

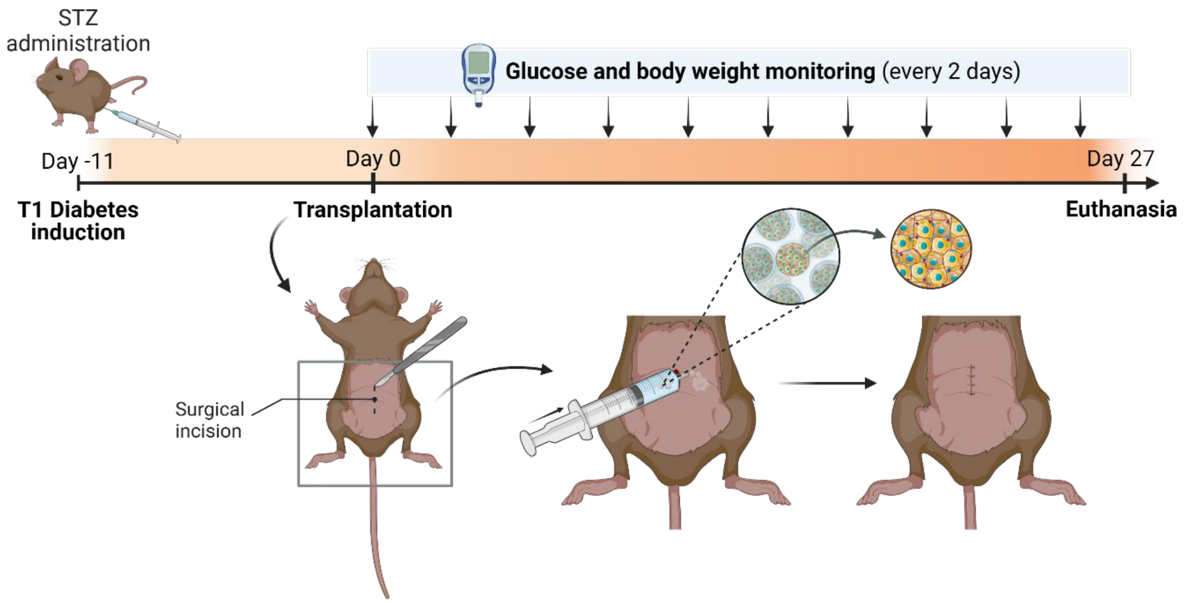

Figure S3. Schematic representation of the *in vivo* experiment and transplantation procedure.

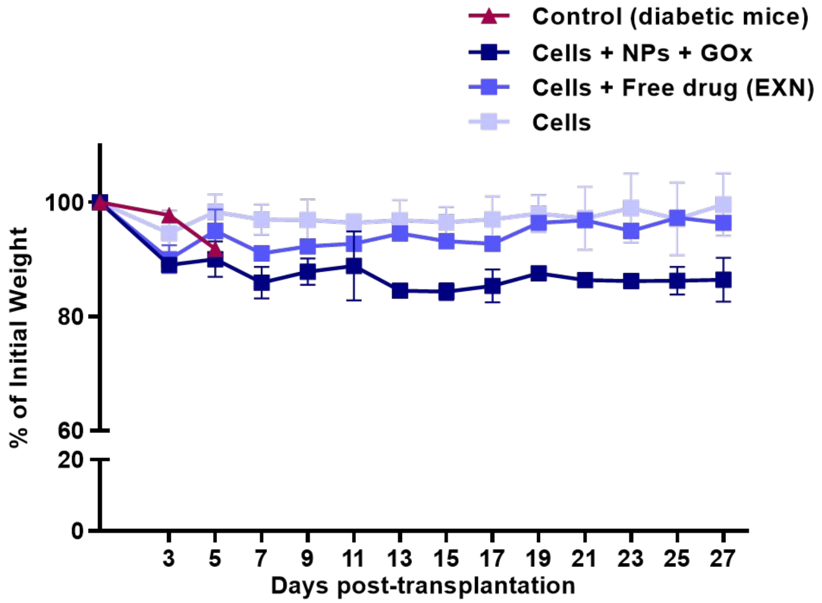

Figure S4. Body weight of streptozotocin-induced diabetic mice after transplantation. Cells were encapsulated either alone (Cells), in combination with EXN-loaded glucose-responsive NPs and glucose oxidase (Cells + NPs + GOx) or in combination with EXN freely dispersed in the alginate matrix (Cells + Free drug (EXN)). Body weight was monitored every two days for 27 days. Values are represented as mean ± SD.
